# Using Health Systems and Policy Research to Achieve Universal Health Coverage in Ghana

**DOI:** 10.9745/GHSP-D-21-00763

**Published:** 2022-09-15

**Authors:** John Koku Awoonor-Williams, Stephen Apanga, Ayaga A. Bawah, James F. Phillips, Patrick S. Kachur

**Affiliations:** a Formerly of Health Service, Accra, Ghana.; b University for Development Studies, Tamale, Ghana.; c Regional Institute for Population Studies, University of Ghana, Accra, Ghana.; d Mailman School of Public Health, Columbia University, New York, NY, USA.

## Abstract

Ghana is positioned to become the first country in sub-Saharan Africa to implement universal health coverage based on nationwide expansion of geographic access through the Community-based Health Planning and Services initiative. This achievement is the outcome of 3 decades of implementation research that health authorities have used for guiding the development of its primary health care program. This implementation research process has comprised Ghana’s official endorsement of the 1978 Alma Ata Declaration, leading to the institutionalization of evidence relevant to the strategic design of primary health care and national health insurance policies and services. Rather than relying solely upon the dissemination of project results, Ghana has embraced a continuous and systemic process of knowledge capture, curation, and utilization of evidence in expanding geographic access by a massive expansion in the number of community health service points that has taken decades. A multisectoral approach has been pursued that has involved the creation of systematic partnerships that included all levels of the political system, local development officials, community groups and social networks, multiple university-based disciplines, external development partners, and donors. However, efforts to achieve high levels of financial access through the roll-out of the National Health Insurance Scheme have proceeded at a less consistent pace and been fraught with many challenges. As a result, financial access has been less comprehensive than geographical access despite sequential reforms having been made to both programs. The legacy of activities and current research on primary health care and national health insurance are reviewed together with unaddressed priorities that merit attention in the future. Factors that have facilitated or impeded progress with research utilization are reviewed and implications for health systems strengthening in Ghana and elsewhere in Africa and globally are discussed.

## Abstract (precis)

Health system implementation research, combined with knowledge management processes, directly contributed to Community-based Health Planning and Services geographic coverage expansion. Research was less deliberately employed for guiding financial access expansion through the National Health Insurance Scheme.

## Teaser

**Key Findings:**

- Coordinated cycles of implementation research, planning, and testing innovations and policy reforms have contributed incrementally to the Community-based Health Planning and Services (CHPS) initiative achieving national scale and improving geographic access to primary health care services at the community level.
- Achieving and maintaining high levels of financial access through the National Health Insurance Scheme (NHIS) has been more elusive, although research and evaluation evidence has also been utilized to support implementation reforms and refinements.
- Although the NHIS contributed to increased use of CHPS services among the poorest, this increase is skewed toward curative care treatment, rather than comprehensive primary health services that include promotive and preventive care.

**Key Implications:**

- Achieving universal health coverage (UHC) in Ghana requires integrating the CHPS initiative and NHIS operations into a fully functional system of care.
- A comprehensive system-learning approach should be adopted in addressing service readiness, geographic availability, and financial access all together.
- Additional research such as a plausibility trial of systems solutions is needed to fully integrate NHIS reform with Ghana’s Roadmap for Attaining Universal Health Coverage, 2020–2030.

## INTRODUCTION

The World Health Organization (WHO) emphasizes that achieving universal health coverage (UHC) requires that all individuals and communities receive the health services they need without suffering financial hardship. This global health objective is formalized by the United Nations Sustainable Development Goal (SDG) target 3.8^1^:

*Achieve universal health coverage, including financial risk protection, access to quality essential health-care services and access to safe, effective, quality and affordable essential medicines and vaccines for all.*

To achieve this goal, WHO’s Thirteenth General Program of Work prioritized expanding UHC to a billion more people worldwide by 2023.^1^ While UHC includes the full spectrum of essential, high-quality services, its foundation is the goal of achieving a strong and resilient people-centered health system with primary health care (PHC).^1^ This focus provided the rationale for Ghana’s Ministry of Health’s (MOH) recent review of its strategy toward achieving UHC that involved developing a national UHC roadmap for strengthening PHC delivery by improving service availability for the population through community health services and the expansion of public health interventions.^2^ In this case study, we review the evidence behind programs and mechanisms that have fostered the utilization of implementation and policy research in Ghana. By gathering and interpreting data at each level of the health care system, this study exemplifies comprehensive health systems implementation research. We describe the state of progress toward expanding geographic and financial access to PHC, with an emphasis on what has been achieved and what remains to be accomplished to reach Ghana’s UHC goals.

We describe the state of progress toward expanding geographic and financial access to PHC, with an emphasis on what has been achieved and what remains to be accomplished to reach Ghana’s UHC goals.

Trends in the coverage of UHC initiatives have progressed over the past 2 decades (Figure). After a period of trial and research in the 1990s, the Community-based Health Planning and Services (CHPS) initiative entered an implementation phase in 2000 that was very gradual until 2009, when implementation accelerated, with levels of coverage eventually approaching UHC goals within the decade that followed. The Figure indicates health insurance coverage, conducted in 2008,^3^ 2014,^4^ 2016,^5^ 2017,^6^ and 2018,^7^ as reported in the national statistics and surveys. The observed trend was less consistent than CHPS coverage trends, despite initially impressive gains in uptake. Based on coverage estimates for women of reproductive age from repeated nationally representative surveys, the proportion of women with valid insurance coverage peaked at roughly 60% between 2014 and 2016 and declined thereafter, hovering around 50%, even though enrollment figures may have been higher.^8^ This article aims to explain these trends and the contrasting coverage of CHPS and the National Health Insurance Scheme (NHIS) over the post-2000 period.

**FIGURE:**
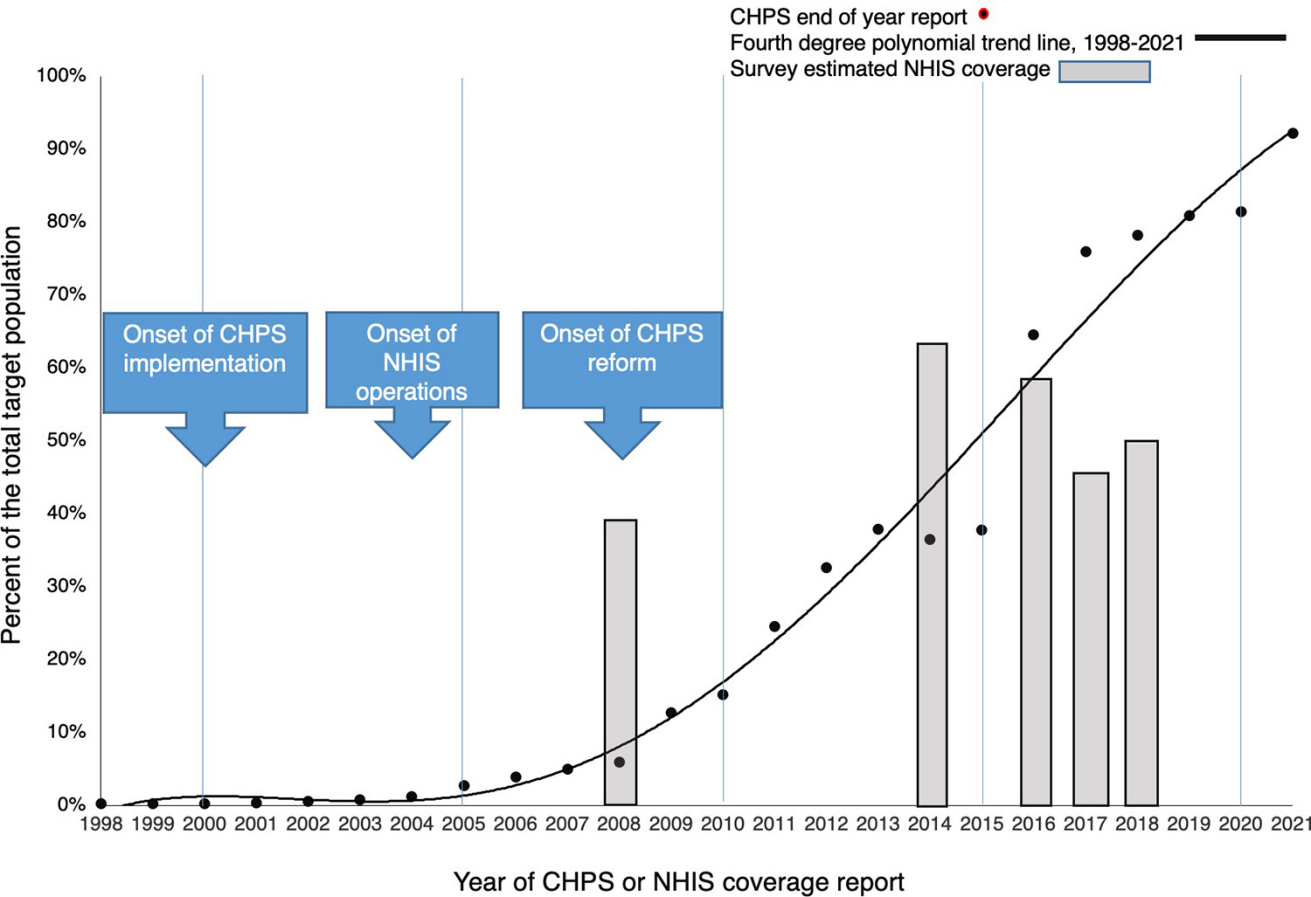
National Coverage of CHPS Compounds and NHIS Active Enrollment, Ghana, 1998–2021^a^ Abbreviations: CHPS, Community-based Health Planning and Services; NHIS, National Health Insurance Scheme. ^a^CHPS coverage information taken from routine National Health Information System data (red dots represent actual numbers of CHPS compounds reported annually through the National Health Information System and the trend line projects the anticipated achievement of full coverage) and NHIS enrollment estimates (blue bars) are derived from nationally representative population surveys.^3^^–^^7^

## THE ROLE OF RESEARCH IN ATTAINING UHC

Ghana’s UHC policy has 2 interlocking components: (1) the CHPS initiative that focuses on achieving geographic access to essential services in people’s communities,^9^^–^^11^ and (2) the NHIS that aims to overcome financial barriers through a national social insurance program.^12^ Because of the systematic application of implementation research evidence to policies that were pursued, CHPS and NHIS are uniquely Ghanaian solutions to the challenges of reaching a culturally diverse, geographically dispersed, and economically disadvantaged population.^13^ Both have been the subject of extensive pilot testing, replication trial, knowledge management, evaluation, reflection, and policy development and reform—to ultimately achieve nationwide UHC.^14^

Ghana’s commitment to achieving UHC commenced with its endorsement of the 1978 Alma Ata Accord.^14^ However, except for launching district hospitals and subdistrict health centers, the country’s economic crises in the 1980s prevented an effective implementation of this accord. While the district hospitals and subdistrict health centers provided essential services, they were intended to receive referrals from more peripheral levels of care because most such facilities were remotely located away from rural households. The MOH responded to this problem by creating a cadre of community-based workers, called community health nurses (CHNs), and deploying these workers as PHC providers at convenient community locations.^15^ However, the core cost of constructing community health posts, providing essential equipment, and providing essential pharmaceuticals for CHNs was unsustainable in most rural districts. CHNs were hired and trained, but because resources for health posts and equipment were inadequate, they were deployed to hospitals and subdistrict clinics where their presence was redundant with other paramedical staff. Moreover, their clinic locations were remote from most households. By the early 1990s, it was apparent to policy makers that “health for all by the year 2000,” as envisioned at Alma Ata, was unlikely to come to fruition soon for most Ghanaians unless reform was pursued.^16^

The geographic accessibility problem was compounded by financial challenges. To sustain the provision of care, the MOH embraced a “cash-and-carry” policy in the 1980s that charged all patients for PHC services. Practical problems with this policy’s provisions prevented families from acquiring essential care. In response to widespread discontent with this policy, an untested “exemption policy” was launched in the 1990s that aimed to provide free services to children aged 5 years and younger. But because this policy lacked the financial support it required, PHC operations were severely disrupted by shortages of essential drugs and supplies. As the year 2000 approached, it was apparent to the policy community that developing geographically accessible services at scale required further evaluation^17^ and a sustainable health insurance program that required careful investigation.^18^^,^^19^ Research stations that had been created by the MOH were commissioned for concerted investigation of solutions to the access and insurance challenges. The Navrongo Health Research Center (NHRC) in northern Ghana was tasked with addressing the access challenge. The Dodowa Health Research Center (DHRC) in Greater Accra Region was assigned responsibility for developing and testing approaches to sustainable health insurance.

By 2000, it was apparent to the policy community that developing geographically accessible services at scale required further evaluation and a sustainable health insurance program that required careful investigation.

## DEVELOPING GEOGRAPHIC ACCESS: THE CHPS INITIATIVE

To generate evidence for guiding discussions on the appropriate strategic design of a program to improve access to PHC services, the MOH requested the NHRC to pilot the deployment of nurses and volunteers to 3 communities based on the WHO “Strategic Approach”^20^^,^^21^ and informed by participatory planning research methods.^22^ A steering committee, chaired by the MOH Director of Medical Services, ensured that research would be embedded into policy planning operations,^23^^–^^25^ with protocols reflecting the need to address strategic questions about the appropriate operational design of community-based PHC services.

The formative pilot and experimental efforts that created CHPS led to a series of implementation studies. When these studies identified problems, research on reform was undertaken that guided the process of improving program functioning. There were 5 critical program components of PHC organization in CHPS that were the direct outcome of implementation research: (1) community engagement; (2) financing; (3) nurse training, deployment, and management; (4) essential equipment procurement and operation; (5) volunteer recruitment, deployment, and management (Table 1).

**TABLE 1.** Pragmatic Observations Associated With the Navrongo Phase 1 Pilot to Increase Access to Primary Health Care Services, Ghana

**Program Component**	**Observation of Pilot Investigators**	**National Policy Outcome**
Community engagement	Traditional and secular leaders support community-based primary health care.^74^^,^^75^	Implementation milestones were documented that included mapping and “community entry.”
	Gatherings (durbars) contribute to community support.^75^	Durbars are recognized as effective means of consensus building for community action.
	Gender problems prevented access to care and women’s inability to realize their reproductive preferences.^76^	Gender development strategies are essential to the implementation of services that depend upon women’s individual agency.
Financing	Service financing: “Trust as insurance:” Payment of “cash-and-carry” fees deferred for episodes of care, based on nurse trust that extended families would eventually reimburse the program.	None.
	Start-up costs: The start-up cost of adding community services to the existing program was less than $10 per capita. Communities will construct interim health post facilities at minimal cost, expediting implementation.^75^	Start-up costs not budgeted until 2009. Delayed response: Table 3.
Nurse training, deployment, and management	Existing nurse training programs were urban based; CHN tended to be unfamiliar with rural residence and norms.	Localized recruitment and training tested and shown to be more effective than centralized training
	Training omitted modules on community engagement.	A 6-month internship and training module added. CHNs completing this certification redesignated as CHOs.
	Ghana has 82 languages: CHN were not always deployed in areas where they spoke local languages.	District-level recruitment, training, and deployment.
	Nurses were ineffective family planning service providers if posted to their ancestral community because residents were concerned about possible breaches in confidentiality if providers were members of informal social networks.^75^^,^^76^	Deployment to localities based on language ability; deployment to home communities was avoided.
Essential equipment procurement and operation	Motorcycles were affordable, but district maintenance capabilities were lacking. Nurses were relocated from clinic residencies where their families were also based.	Logistics development was organized. Nurses were trained in motorcycle use and basic maintenance. Fuel delivered to nurses during supervisory outreach rounds.
	Essential drugs: Nurse deployment accelerated the volume of primary care encounters, depleting pharmaceutical supplies.	Logistics reform factored in acceleration of supply requirements.
Volunteer recruitment, deployment, and management	Volunteer recruitment and deployment is feasible. Volunteers were effective in providing outreach to men.	Volunteers recruited in conjunction with CHO deployment; CHO consigned supervisory and community engagement functions.
	Supervision and management: Community-based care amplified the need for supervisory outreach.	District CHPS coordinators added to the CHPS staffing structure with CHO, CHVs, and CHMCs.

Abbreviations: CHMC, community health management committee; CHN, community health nurse; CHO, community health officer; CHPS, Community-based Health Planning and Services; CHV, community health volunteers.

As of 2021, the national implementation of CHPS has reached 6,277 of the 6,809 targeted service catchment areas. This achievement spans over 2 decades of implementation effort.

### Phase 1: Developing a Community-Based Strategy

Advocacy, based on international agency initiatives elsewhere, suggested contrasting approaches to community-based operations. UNICEF’s experience with volunteer-based services in the Republic of Benin was translated into the Bamako Initiative,^26^^,^^27^ which involved convening community health committees for managing revolving accounts to sustain the financing of essential drugs. Unpaid volunteers were recruited and trained to provide primary care and referral services. Several international commentators viewed the Bamako Initiative as an affordable means of achieving health for all.^28^ Ghana’s experience with volunteer initiatives in the 1980s raised questions about the quality of care that volunteers were capable of providing as well as reasons to doubt the sustainability of unpaid volunteer approaches.^29^^,^^30^ In response to the view that paid professional workers were essential to the provision of PHC, the MOH launched a program that involved training CHNs for 18 months. However, the absence of evidence on the feasibility and effectiveness of CHN deployment at the community level could not justify investment in their training.

A pilot intervention was developed to assess the feasibility of volunteer versus CHN placement in 3 communities in Kassena-Nankana district in the Upper-East Region (UER) of Ghana. Operational problems were taken to community committees for discussion. All 3 communities expressed an interest in having resident nurse access and a willingness to assemble volunteer teams for constructing interim facilities using traditional methods and materials with the understanding that their effort to create locations for services would be rewarded with the posting of a resident nurse (Box 1).

BOX 1 **Community-Constructed Interim Facilities in Ghana**

**Figure:**
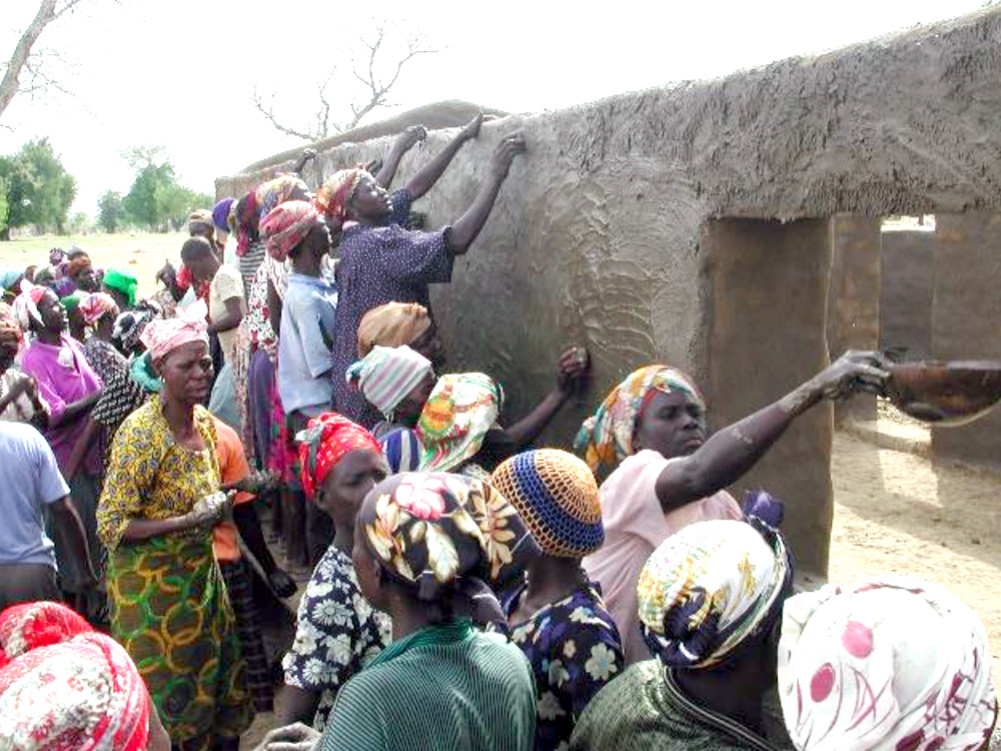
The costly construction of permanent facilities where resident nurses can live and provide care is critical to launching community-based primary health care services. Participatory appraisal of this problem generated evidence that communities would contribute volunteer labor, material, and traditional methods for interim facility construction that would be replaced with permanent structures when financing became available. Initially demonstrated in Navrongo, and replicated in Nkwanta, community construction was adopted as a routine Community-based Health Planning and Services (CHPS) component when GEHIP demonstrated procedures for routinizing the method as a means of accelerating the launching of lifesaving CHPS. © 1996 James Phillips/Population Council

Volunteer deployment was also piloted, generating experience with the process of recruitment, training, and supervision for community health volunteers. The apparent feasibility of both strategies contributed to policy commitment to proceed with community-based program planning. The relative efficacy of the pilot was assessed as a phase 2 district experiment that focused on assessing health and demographic endpoints^17^ that have been extensively disseminated elsewhere.^31^^,^^32^

Practical experiential learning from the trial that profoundly affected national operational policy had garnered less attention in the formal literature on research outcomes. The phase 1 pilot was limited to 3 communities spanning only 18 months of qualitative participatory appraisal of community reactions to services for eliciting grassroots advice in strategic planning.^22^ Considerable experience was nonetheless gained that was relevant to addressing practical challenges associated with community engagement, worker deployment, and financing and sustaining operations (Table 1, column 1). To clarify operational strategies to be tested in a phase 2 experiment, practical learning was shared with the MOH oversight team (Table 1, column 2), which had a lasting impact on CHPS planning, implementation, and policy (Table 1, column 3).

### Phase 2: Testing the Strategy

Despite important operational lessons that phase 1 provided, the efficacy of volunteer versus CHN deployment in the community could not be resolved by a 3-community pilot. A district-wide trial was required with study arms: nurse only, volunteer only, and combined nurse and volunteer deployment. These conditions could be tested relative to the effectiveness of care limited to subdistrict and hospital clinics.

This experiment, referred to as “the Navrongo Experiment,” was configured by assigning each of the 4 subdistricts of Kassena-Nankana district to one of the worker deployment conditions, whereby all communities were exposed to 1 or both of the conditions.^17^ Overall community-based care was found to be affordable (US$8.72 per capita per year) and the incremental cost of CHPS implementation—incorporating both resident nurses and volunteers—was only US$1.92 per capita. Work routines, supervisory arrangements, and other important administrative details provided an evidence base for the GHS to plan national scale-up.^9^ Most importantly, the basic staffing parameters of the national community-based program were clarified (Table 2).

**TABLE 2.** Pragmatic Observations and Policy Outcomes Associated With the Navrongo Experiment to Increase Access to Primary Health Care Services, Ghana

**Program Component**	**Observation of Pilot Investigators**	**National Policy Outcomes**
Nurse training, deployment, and management	Nurse services have a pronounced impact on child health and survival in experimental cells where community-based nursing was functioning.	The combined configuration of worker deployment was adopted as national policy.^9^
	Nurse deployment, without volunteer support, had no impact on family planning use or fertility.^31^^,^^76^	
	Combining nurse deployment with volunteer-supported community engagement resulted in significant effects on child survival, family planning use, and fertility.^32^^,^^77^	
Volunteer recruitment, deployment, and management	Volunteer deployment had no impact on child health or survival and no independent effect on family planning or fertility.^32^^,^^78^	Volunteer deployment was a dual cadre initiative whereby volunteers were deployed to support the services of nurses but were not primary service providers.

### Phase 3: Setting the Stage to Scale Up the Strategy

In 1998, the MOH convened a National Health Forum with all district, regional, and national directors of health services to build consensus for scaling up the Navrongo Experiment generated evidence that is summarized in Tables 1 and 2. Consensus could not be reached as debate ensued about the feasibility of transferring the Navrongo system to other districts owing to diverse environmental, cultural, and economic circumstances (Box 2).

BOX 2 **The Staffing Outcome of the Navrongo Initiative: Paid Nurses and Volunteers for Optimizing Community Engagement**

**Figure:**
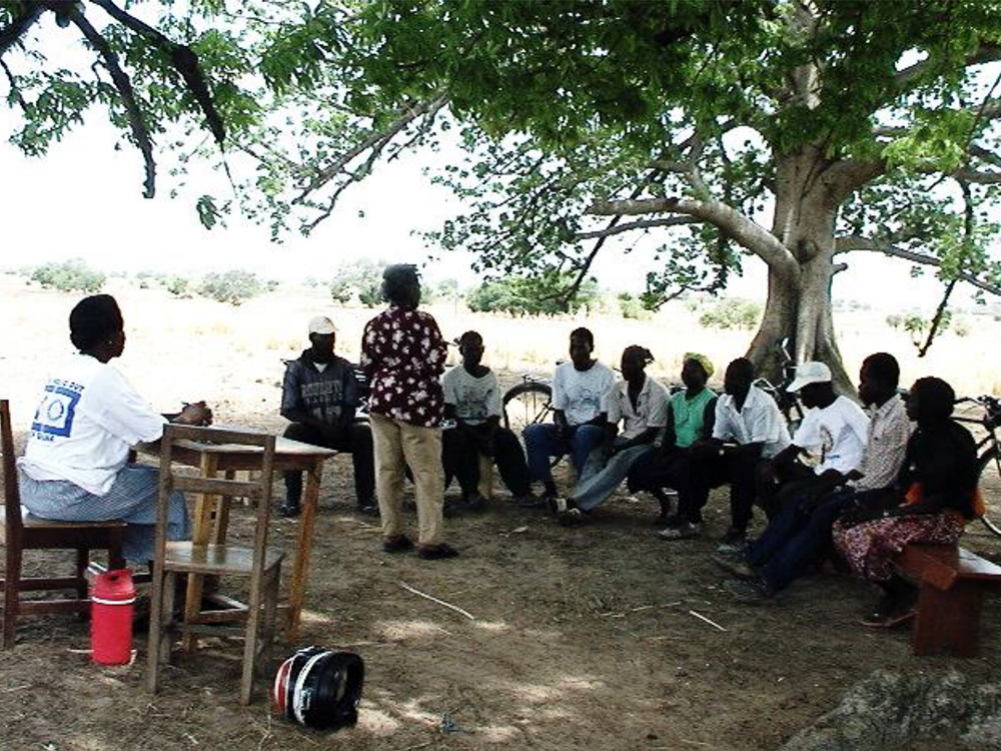
Results from the Navrongo experiment showed that volunteer deployment had no impact as a stand-alone scheme. While nurse deployment had major child survival effects, family planning and fertility results depended upon community health nurses and volunteers talking to men in the community about family planning. The joint nurse and volunteer combined staffing model was adopted as national policy. ©1996 James Phillips/Population Council

The Nkwanta District Health Director from the Volta Region of Ghana offered to test the transfer of the Navrongo approach. A 1-year reprieve on action in Navrongo was agreed upon, whereby Navrongo pilot activity was transferred to Nkwanta district to be replicated and adapted.

Results of the Nkwanta replication process are summarized in Table 3. National policies for staffing, capacity building, scale-up, operational dissemination, support systems development, and knowledge generation and management were outcomes of the Nkwanta initiative. Initially viewed as a 1-year demonstration, Nkwanta became a sustained resource, whereby teams from various districts across the country undertook peer learning exchanges, which resulted in scaling up CHPS operations nationwide for a decade.

**TABLE 3.** Pragmatic Observations and Policy Outcomes Associated With the Nkwanta Replication Process, Ghana

**Program Component**	**Observation of Pilot Investigators**	**National Policy Outcomes**
**Staffing**		
Nurse and volunteer deployment and management	Replication of the Navrongo model was feasible and affordable, although cultural heterogeneity required greater focus on community engagement and community-based decentralization than in Navrongo.	A 1999 National Health Forum adopted CHPS as national policy.^9^ The combined configuration of worker deployment was replicated. Implementation results legitimized national implementation policy commitment.^79^^,^^80^
**Capacity building**		
Training	Within district, implementation required implementation-based team demonstration to supplement technical training.^80^	Results led to established systems of CHPS-specific operational guidelines and orientation for CHNs before and during deployment.
Within-district scale-up	Peer learning was essential to community-based replication of CHPS operations. Team exchanges developed systems thinking at each level of the operation.^80^	Intra-district teams peer exchanges and CHPS operations orientation among facilities was instituted.
Between district dissemination of operational learning	Although six essential milestones were critical to disseminating CHPS,^79^^,^^81^^,^^82^ their replication required participatory learning. Didactic training and documentation of approaches were helpful but insufficient. ^82^ Eight lead districts were trained in peer learning processes.^83^	District teams’ peer exchanges and CHPS operations’ orientation among districts with CHPS centers of excellence was instituted in other districts (e.g., Birim North, Abura-Asebu-Kwanakese, and Juabeso Bia districts).
**Support systems learning**		
Supervision and management	Supervision was focused on visiting their assigned communities for collaborative problem solving.^84^	Regular subdistrict health teams and DHMT quarterly supportive supervision were established.
Logistics and supply	Through the support of another project, the Ghana Essential Medicines Initiative, critical supplies and medicines were made available at CHPS locations that meet the needs of the disease’s profiles of the zones.	Basic essential supplies and equipment list was developed for CHPS operations and donor support for logistics was marshaled.
**Research and knowledge management**		
Within district learning processes	Durbars that involved participatory leadership exchanges could be used to spread community-level understanding of CHPS and commitment to scale up.	Developed manuals and guidelines for community engagement and understanding of CHPS and community durbars regularized.
Scaling up learning	Experiential learning through participation and observation was an effective means of building senior official knowledge of implementation processes and results.	Annual national health fora and senior managers conferences became focal points for CHPS operations and discussion and performance evaluation.

Abbreviations: CHN, community health nurse; CHPS, Community-based Health Planning and Services; CHV, community health volunteers; DHMT, district health management team.

The Nkwanta initiative resulted in national policies for staffing, capacity building, scale-up, operational dissemination, support systems development, and knowledge generation and management.

### Phase 4: Scaling Up CHPS

Although policy pronouncements endorsed the scale-up of CHPS by the year 2000, initial progress was gradual for a decade (Figure 1). Rapid scale-up of CHPS ensued in 2009, however, with national coverage subsequently expanding until most districts were saturated with functioning zones within the following decade. By 2019, this saturation was a time of consolidation of gains, with policies directed to improving coverage by splitting zones and dispersing workers. Thus, by 2021, original coverage objectives had been met, with new goals defined by the need for CHPS refinement and following the creation of new administrative areas. We consider each of these eras, in turn.

Navrongo and Nkwanta field operations were used to expand national implementation capacity through field-based demonstration activities for visiting implementation teams. Rather than focusing on individual training, the orientation process was designed to develop team implementation capacity.

Participating districts were invited to assemble a complete CHPS implementation team comprised of district health management team representatives, a subdistrict supervisor, and at least 2 community health nurses called community health officers (CHOs) who had received additional in-service training and were scheduled to implement CHPS services. Participants observed the process of implementing the 6 Nkwanta implementation milestones (1) mapping, zone planning, and initial community leadership engagement; (2) community staff orientation and training; (3) local governance development; (4) community facility construction; (5) logistics mobilization and nurse deployment; and (6) volunteer mobilization, to equip implementation teams to establish at least 1 functioning zone where Nkwanta-like CHPS start-up and management procedures could be demonstrated. Equipped with this capability, participating teams could replicate the Nkwanta zone-by-zone process of rolling out CHPS as resources, capabilities, and community commitment emerged. This approach, termed “guided diffusion” was intended to foster the spread of CHPS among communities at the district level, even before national revenue was available to cover the district-level cost of starting the program.^33^

### CHPS Reform: A Renewed 4-Phased Process of Implementation Research

The participatory orientation program had an immediate impact. By the end of 2008, 92% of all CHPS coverage was associated with implementation activities in 32 districts where implementation teams had experienced the team-based orientation visits. However, implementation in other districts was slowed by leaders’ lack of understanding of implementation milestones and the absence of resources for start-up costs. Reviews of capacity building,^34^ implementation leadership,^35^ and community engagement in districts that were not progressing with scale-up attested to the need for CHPS implementation reform. When elements of a possible reform package were clarified by a field review of CHPS implementation challenges,^35^ the GHS launched a trial of reform implementation in the Upper East Region (UER). Conducted in 2010–2015, this trial tested strategies for implementing elements of the 6 WHO health systems strengthening pillars.^36^

Known as the Ghana Essential Health Interventions Program (GEHIP), this initiative, located in 4 districts, commenced with a participatory community appraisal of the goals of national CHPS reform. Comprising a new phase 1 component of a research agenda, this investigation highlighted practical means of addressing problems that were hampering CHPS scale-up. Followed immediately with a 4-district trial of health systems strengthening activities, GEHIP tested means of involving communities in rapid implementation activities,^37^ improving the provision of emergency care,^38^ and reforming budgeting and financial mechanisms. These efforts expanded CHPS coverage from 20% to 100% in 4 years in treatment areas within the UER.

GEHIP applied lessons from the original phase 1 Navrongo pilot for the process of convening community-engaged construction of interim community care facilities. Rather than limiting action to 3 communities, however, GEHIP applied this strategy to 4 districts, leading to rapid expansion of CHPS coverage in treatment districts. GEHIP also addressed the problem of fragmentary emergency referral services with a community-engaged program of logistics support, emergency communication, and acute care development.^37^^,^^38^ Comparison district increases in coverage were also pronounced, but coverage impacts were half that of GEHIP.^37^^,^^38^ Mortality declined throughout the UER during the GEHIP period, but observed declines were significantly greater in treatment districts than in comparison districts.^39^

In response, several implementation lessons were adopted by the GHS. However, the continuing need for system learning led to further study and action. With support from the Korean International Cooperation Agency, GEHIP was expanded to cover all 13 UER districts to create a region of excellence for UHC achievement.^40^ A concomitant transfer experiment was launched to test the replicability of GEHIP in 2 districts each in the Northern and Volta regions. This ongoing process of operational learning is termed the Program for Strengthening the Implementation of the Community-based Health Planning and Services Initiative in Ghana or CHPS+.^41^ Taken as a set of learning activities, CHPS+ incorporates knowledge management operations as an integral component of its embedded system of project management, research, and utilization (Table 4).^42^ As Table 4, column 3 shows, CHPS+ has demonstrated that GEHIP methods for fostering community-driven construction of interim facilities could be replicated, thereby minimizing start-up costs and delays that arise from expensive and complex construction procurement procedures (Box 1).^43^

**TABLE 4.** Pragmatic Observations and Policy Outcomes Associated With Monitoring the Scaling Up Process

**Functional Component**	**Observation of Investigators Regarding Operational Challenges That Emerged With Time (2000–2008)**	**Interventions and Findings From GEHIP That Were Replicated by the CHPS+ Initiative**
**Staffing**		
Nurse and volunteer deployment and management	Volunteer deployment drifted from its original community engagement and male outreach component. Nurse deployment became increasingly focused on facility-based service delivery with less staff effort directed to field activities and community outreach.^85^^,^^86^	Volunteers could be focused on supporting “integrated management of childhood illness” services of CHO, home visit, and male outreach. Supervisory outreach, outreach scheduling, and management capacity could be developed through demonstration and peer learning.
	Nurse deployment was delayed by the absence of revenue, plans, or budgetary provision for startup costs. In particular, the construction of community health posts was delayed. Without facilities for residential nurses posting, community-based services cannot commence.	Community leaders were committed to solving this problem by mobilizing volunteer construction of health posts. Grassroots politicians gained an understanding of the popular support for launching CHPS, using development revenue as a seed fund for starting construction.
**Capacity building**		
Training	Team engagement for peer learning that was the hallmark of Nkwanta dissemination success was abandoned, mainly because donor support for exchanges ended and budgets for this activity were absent. Inservice training focused on technical issues rather than evidence based on quality assurance research.	Re-introducing and institutionalizing peer exchanges and sharing practical lessons learned on CHPS operations among intra- and inter-district, as well as inter-regional teams could lead to success in CHPS implementation.Use of implementation research to guide and inform CHPS operations could lead to evidence-based decisions and quality of CHPS services.
Within-district scale-up	District leader tended to link scale-up to the provision of funds for construction. Community engagement and community volunteer interim construction waned with time.^35^	Effective community and stakeholder engagement could lead to provision of CHPS infrastructure. Traditional methods of community mobilization for health actions engender community support for CHPS and availability of interim community-led construction of CHPS facilities.
Between-district dissemination of operational learning	Opportunities for district leadership to learn about practical strategies for within-district CHPS scale-up because implementation learning was impaired by over-reliance on documentation, didactic training, and meetings.^35^ Experiential field demonstration was lacking.	Each region should have a “systems learning district,” where practical implementation planning and action can be demonstrated. CHPS+ SLD established district demonstration capabilities that merit national replication. If linked to national fora and performance reviews on CHPS, SLD could amplify understanding of CHPS implementation for district teams and leadership.
**Support systems learning**		
Supervision and management	Supervision was found to be effective if linked to outreach activities and engagement with workers in CHPS zones.^87^ But supervisors were also functioning as sub-district paramedics who relied upon NHIS reimbursement fees. This pattern of costing and compensation contradicted the need for field work and rewarded instead time spent in clinic locations.^88^	Field and on-the-job coaching and mentorship of CHPS nurses could be more effective in motivating and scaling up CHPS. The importance of good supportive supervision leads to better capacity of CHPS nurses.

Abbreviations: CHN, community health nurse; CHO, community health officer; CHPS, Community-based Health Planning and Services; CHPS+, Program for Strengthening the Implementation of the Community-based Health Planning and Services Initiative in Ghana; GEHIP, Ghana Essential Health Interventions Program; NHIS, National Health Insurance Scheme; SLD, system learning districts.

## EXPANDING FINANCIAL ACCESS: THE NATIONAL HEALTH INSURANCE SCHEME

Out-of-pocket payments and the uncertainty around them have long been recognized as barriers to accessing needed health services in low- and middle-income countries, including Ghana. The application of user fees was commonly recognized as 1 factor exacerbating socioeconomic inequities in the uptake of health services.^43^ Before 2000, Ghana had addressed some of these through targeted user fee exemptions for specific groups including pregnant women, young children, health workers, patients with a limited range of conditions (e.g., TB, leprosy, mental illness), and the extreme poor. These exemptions were not consistently applied and therefore failed to resolve financial barriers or achieve equity.^44^^,^^45^ In some cases, research showed that health care providers and facility managers relied on user fees to maintain operations and did not want to apply the exemptions as required.^46^ There had also been limited experience with social health insurance programs for formal sector employees and others able to purchase coverage in the private sector.^47^ These efforts did not include the vast majority of Ghanaians who subsist on the informal economy and were not “pro” poor. To address these groups, small-scale public and private sector mutual health organizations arose in several settings. These efforts to establish a community-based health insurance scheme were limited in scope—reaching only 1% of the population,^48^ and their terms and coverage were not well coordinated and could not be used outside their limited geographic areas.^49^ Despite these developments, the highly unpopular system of user fees continued to dominate, and abolishing it became a political issue in the national elections of 2000.

The NHIS was established by an Act of Parliament and signed in September 2003 “to secure the provision of basic healthcare services.”^50^ The legislation also established the National Health Insurance Authority (NHIA) to implement a national health insurance policy, beginning in 2005. The move was widely celebrated as one of the very first efforts by a sub-Saharan African country to provide social health insurance for all.^51^ A main objective of the NHIS was to reduce financial barriers by providing enrollees with a standard package of health services intended to cover 95% of all health care needs. The NHIS benefits are portable and can be accessed at any accredited health facility nationwide. The NHIS is financed by a levy on value-added taxes, contributions to the Social Security and National Insurance Trust (SSNIT) from formally employed workers, individual premiums paid by informally employed members, and returns on investments made by the National Health Insurance Fund (NHIF) through Parliamentary allocation and donor contributions.

A main objective of the NHIS was to reduce financial barriers by providing enrollees with a standard package of health services intended to cover 95% of all health care needs.

To participate as beneficiaries, individuals and households must apply in person and pay an initial registration fee and annual premiums. Some groups are exempted from paying the annual premiums, including SSNIT contributors and pensioners, as well as some groups subject to user fee exemptions already: children younger than age 18 years (whose parents enroll), persons older than age 70 years, those with physical disabilities or mental illnesses, pregnant women and newborns (since 2008), and persons and households designated indigent by the Ministry of Social Welfare.^53^ However, despite these exemptions, some still face some form of financial hardships. For example, except for pregnant women and newborns who have immediate access to health care services after enrollment, the rest of those exempted have to serve a waiting period of 3 months as new registrants or 1 month for defaulting in paying their annual premiums.^53^ The annual premium charged can vary according to a person’s income or wealth, but a standard rate is most often applied.^54^

The proportion of the Ghanaian population enrolled in NHIS increased rapidly in its early years, growing from 25% to 42% between 2006 and 2007.^55^ However, the proportion who maintained coverage after initial enrollment, especially among those required to pay annual premiums has consistently lagged behind enrollment figures. Some individuals and families could not afford the annual premiums, while others prioritized other household expenses, planned to wait until they needed health care, or simply concluded that NHIS provided too little coverage to families to justify the expense of annual renewal.^56^ Evidence also suggests that a substantial portion of individuals were unaware of when their coverage expired, or the need to renew through annual premiums.^57^ In the face of declining NHIS coverage, research also indicated that the poorest individuals in remote areas were often the least likely to enroll or renew.^58^ Despite these challenges, there is evidence that the NHIS is reducing out-of-pocket payments for health services.^58^^,^^59^ Yet, the extent of this contribution to financial access has been less than expected.^59^ In 2008, a Maternal Exemption Policy was added to the NHIS. The idea is to eliminate premiums for women who were pregnant and to automatically cover neonates to remove any coverage gap between birth and registration.^60^

Accredited health facilities that provide covered services to individuals with valid NHIS credentials can submit claims for reimbursement to recover the cost of the services provided. However, there have been challenges with ensuring timely remittances in response to these claims. At times, the NHIF has not been replenished sufficiently quickly or completely to manage all of the claims. Delays of 6 to 12 months or more have become commonplace.^61^ At the start of the scheme, NHIA required providers to submit itemized billing without specification of standardized fees. This process was cumbersome and contributed to the inefficient processing of claims; it also resulted in wide-ranging claim amounts for the same services. By 2008, the NHIA had introduced the Ghana Diagnosis Related Groups (GDRG) schedule that standardized fees for specific conditions at all levels of service.^62^ This created some additional challenges, including incentives for providers to bill for the maximum allowable, whether a full range of services was actually provided to each client.^63^ An investigation in 2010 demonstrated that the GDRG had failed to contain costs, particularly for outpatient services, which accounted for the majority of claims.^62^

Over the past decade, the sustainability of the NHIS has raised concerns for the government mainly due to the rising volume of claims and the associated unrecovered cost. As the volume and cost of claims increased, replenishment of NHIF was slower than expected.^64^ In 2010, as a cost containment strategy, the NHIA began exploring a capitation payment system, through which providers would be afforded a single fee per individual, meant to cater to the whole range of covered services that the beneficiary would be expected to require over the course of the year.^65^ The system was piloted in the Ashanti Region in 2012; however, shortly after, political enthusiasm waned, and it was never expanded nationwide.^66^

A major reform of the NHIS is that it provides a single benefit package to everyone nationwide who registers regardless of employment, income, or age. The package is intended to cover the majority of diseases and conditions that afflict Ghanaians^67^ and includes outpatient services, including diagnostic testing and operations; all maternity care services, including cesarean deliveries; most inpatient care, including surgeries, specialist services, and hospital accommodations; oral health services; emergency care; and all drugs on the essential medicines list.^54^ Some major surgeries, cancer treatments, and cosmetic surgeries are excluded. Some of the challenges and barriers experienced have included: adding to the demands on health infrastructure and human resources, long delays in reimbursement for covered services which affected the financial sustainability of service provision at health facilities, the costs to the population of enrolling and renewing, required copayments, and the lack of coverage for some specific services, treatments, and procedures.

A major reform of the NHIS is that it provides a single benefit package to everyone nationwide who registers regardless of employment, income, or age.

A particular challenge for achieving UHC for preventive health components of PHC is that many basic preventive services are not specifically covered.^61^ Initial plans earmarked at least 10% of the NHIF for preventive services, but this has yet to be realized. Instead, in the early years, NHIF covered the country’s copayment for immunizations procured through Gavi, the Global Vaccine Alliance.^68^ As a result of this omission, CHPS compounds—which provide the majority of preventive services and a critical amount of basic curative care—have not benefited from the NHIS to the extent of subdistrict health centers and district hospitals. In fact, some district managers have deliberately staffed CHPS compounds to be able to provide a greater volume of reimbursable curative care, potentially neglecting the CHPS initiative’s fundamental preventive and community health focus.^61^ Moreover, reimbursement schemes fail to address the need for CHPS outreach and community mobilization costs. The focus on care reimbursement has had a dysfunctional tendency to divert CHPS from its community-oriented care origins by emphasizing instead its role as a community-based clinical program for curative care.

A 2016 presidential technical committee reviewed the challenges associated with NHIS implementation since its inception. While this review recommended that the scheme be restricted to a compulsory primary health care and maternal and child health care provision,^69^ thereby enabling the scheme to be more focused and serve as a vehicle for UHC, multiple operational problems persist.

## DISCUSSION

It is the goal of UHC that all people obtain the health services they need without risking financial hardship arising from unaffordable out-of-pocket payments. This involves quality health services coverage ranging from health promotion to prevention, treatment, rehabilitation, and palliation as well as coverage with a form of financial risk protection. The role of the CHPS initiative toward achieving UHC in Ghana cannot be overemphasized. Whereas the NHIS has a wide range of service packages that it pays for, these are concentrated more on treatment to the detriment of the other service coverage areas of UHC especially health promotion and prevention including outreach services which are core functions of CHPS. Current evidence suggests that the existence of the NHIS has led to increased use of the CHPS services by the poorest who are insured.^70^^,^^71^ However, under the current dispensation of the NHIS, this increase is only skewed toward treatment or curative care, which could negatively impact Ghana’s roadmap to achieving UHC. Even though the determinants of enrollment into the NHIS have been extensively researched, empirical evidence to determine the effect of a strengthened health system, especially the CHPS initiative, on NHIS enrollment deserves further research. This should be complemented with a new round of implementation research that addresses complete UHC integration of the NHIS with CHPS. The complementary ability of a strengthened PHC system such as CHPS and a social pool health financing mechanism such as the NHIS with a focus on PHC could serve as the best pathway toward achieving UHC.

Thus, with the development of a national UHC roadmap, continuous efforts at ensuring that Ghanaians have timely access to quality health services irrespective of their ability to pay at the point of use should be a national priority. Key strategies in operationalizing the UHC roadmap must therefore include increasing NHIS enrollment and keeping members active and making CHPS functional throughout the country.

## LESSONS LEARNED

UHC in Ghana is imminent. This achievement is the consequence of 4 decades of policy formation, public investment, and action that has been grounded in Ghana’s commitment to the 1978 Alma Ata Declaration and later emphasized by Astana 2018. Research has been critical to implementing strategies that work. When scaling up rapidly progressed without research grounding, several operational problems arose. Fortunately, the process has also led to 7 key learnings based on what has worked well.

Research has been critical to implementing strategies that work toward achieving UHC.

1. No single project or initiative has had UHC as the outcome. Rather, Ghana has instituted a process for sequential research to build on accumulated learning. Progress achieved will not end the continuous learning process; research for developing PHC will continue. The process of sequential implementation learning has been sustained by CHPS, with an apparent effect on the sustainability of CHPS itself.^65^^,^^66^ In contrast, the NHIS was launched based on pilot implementation. Research that ensued focused on diagnosing operational problems rather than testing implementation improvements. Results indicate a need to reverse research priorities: CHPS should have a new research agenda focused on problems and quality of care, and the NHIS should prioritize implementation research for achieving sustainable mass coverage as well as complete integration of NHIS and CHPS.
2. The CHPS learning process constitutes an example of embedded implementation science,^23^^,^^72^^,^^73^ while NHIS research has been planned, conducted, and disseminated by researchers. CHPS research directions and findings are owned by the government GHS leadership structure, while NHIS research is external to the system that it aims to improve. The CHPS integrated and comanaged approach maximizes prospects that research outcomes will serve as program implementation inputs.
3. Key policy-relevant evidence has been institutionalized through deliberate knowledge management—through active and ongoing engagement between policy makers, implementation authorities, and researchers—and not merely passively disseminated. Research has used mixed methods, always with a theoretical grounding that permits rigorous inference, and scaling up has been the subject of research, not just a decree at the end of each project phase or research episode. Carefully designed mixed method research has provided valuable insights into NHIS functioning, coverage, and impact.
4. External financing has been critical to the conduct of NHIS research but not controlling. Multiple donors have been involved in NHIS research, but none directed or financed operations on these initiatives. In general, the NHIS research has benefited from avoiding cooperating agency implementation activities. Several donors that have been engaged in CHPS implementation have often used their own systems of operations and procurement that have been dysfunctional to the GHS administrative procedures, processes, and ownership.
5. CHPS and NHIS research operated at all levels of the system, unified by a system-thinking perspective. In both the NHIS and CHPS development domains, political leadership engagement contributed to UHC's impact when the research system policy system engaged all levels of the political system. All aspects of the intervention are integrated: no particular modality, service strategy, or technical input has dominated the research operation; for the health insurance system, all levels of the health care operation have been the focus of research.
6. Learning takes time, especially in complex systems contexts. Projects can have artificial timelines; processes must be sustained beyond the duration of any particular project. Even as UHC approaches, the need for system learning continues. Both the NHIS and CHPS have complex implementation challenges to address in the future.
7. For both the NHIS and CHPS, failure and setbacks have been the focus of learning and corrective action rather than an outcome that terminates investigation. Diagnostic research has been a continuous component of the learning process. Problems that persist will now be the subject of forthcoming investigation and action. Achieving UHC involves integrating the CHPS initiative and NHIS operations into a combined fully functional system of care. While the concomitant presence of CHPS with NHIS represents progress to this end, operational details of this partnership merit further implementation research. Cost recovery arrangements support the provision of CHPS clinical services, but inadequately address links between NHIS reimbursement mechanisms and CHPS outreach, preventive services, and community engagement operations. Accessible services will fail to achieve UHC if achieving financial accessibility remains elusive. For this reason, a comprehensive system-learning approach should not be limited to addressing service readiness, geographic availability, and financial access in isolation.

For the NHIS and CHPS, failure and setbacks have been the focus of learning and corrective action rather than an outcome that terminates investigation.

## FUTURE DIRECTIONS

Further systems trials and investigations are needed that aim to develop a fully functional UHC system in trial districts, using Ghana’s recently implemented CHPS+ project with its functioning PHC system as a platform for implementation research on developing and testing NHIS reform.

What would such an investigation entail? To progress toward UHC, a new phased implementation research initiative is needed that builds on the successes of the CHPS process of sequential investigation and that addresses the deficiencies of the NHIS program highlighted in insightful studies. This research program should use the informative strategy of NHIS research on diagnosing operational problems to investigate CHPS quality and operational problems associated with functionality. The following 4 overlapping phases could be pursued.

To progress toward UHC, a new phased implementation research initiative is needed that builds on the successes of past research and addresses deficiencies.

1. A strategic crossover approach would commence with a rapid qualitative systems appraisal of health care worker, supervisor, and district manager reactions to investigator-posited improvements in the total system of care, focusing primarily on possible NHIS reform.
2. Based on the lessons learned, stakeholders could launch a new plausibility trial in districts that have achieved advanced coverage with CHPS operations but where possible flaws in the regimen of CHPS services require investigation.
3. In response to phase 2 continuous observation of the district trial, knowledge management and systems research inference could be managed in conjunction with replication activities, designed to assess the scaling up potential of the NHIS reforms that are under investigation.
4. When phase 3 scale-up procedures have evidence to guide action, a national reform of NHIS and CHPS could proceed with evidence to guide the national reform process.

The role of research in guiding UHC remains crucial and would evolve as health system changes and programs mature over time. Achieving UHC will require incremental operational learning and a flexible research and program paradigm to ensure that findings from embedded IR can be incorporated into policy implementation processes as they arise, even when the coverage of the NHIS and CHPS is complete. Research strategies that have contributed to CHPS and NHIS development in the past merit review and utilization for addressing UHC development needs in the future.

## Notes

**Peer Reviewed**
